# A beam-direction cross-vessel metrology method and error analysis for the ptychography setup at HEPS ID09

**DOI:** 10.1107/S160057752600634X

**Published:** 2026-07-16

**Authors:** Ruiying Liao, Zina Ou, Haihan Yu, Xuan Wang, Jie Dong, Aiyu Zhou, Wenkai Kang, Liang Zhou, Shanzhi Tang, Ming Li, Weifan Sheng

**Affiliations:** a Multidisciplinary Research Center Institute of High Energy Physics of the Chinese Academy of Sciences Beijing People’s Republic of China; b College of Nuclear Science and Technology University of Chinese Academy of Sciences Beijing People’s Republic of China; Tohoku University, Japan

**Keywords:** metrology method, error analysis, interferometers, ptychography setup

## Abstract

A metrology method dedicated to position monitoring and correction for the ptychography setup at the High Energy Photon Source (HEPS, Beijing, China) beamline ID09 is proposed. The sophisticated design of the Invar interferometer metrology frame ensures optimal performance within the compact environment between the focal spot and the sample setup. The measurement method and error analysis are described in detail. The sample setup achieves a positional stability of less than 2 nm RMS from 1 Hz to 500 Hz. The 12.3 nm reconstruction resolution of 2D ptychography at 12.4 keV demonstrates that the interferometer metrology strategy is available.

## Abstract 2

A beamline-direction cross-vessel metrology method and error analysis for ptychography setup at HEPS ID09 is proposed and implemented. A 12.3 nm reconstruction resolution of ptychography demonstrates the validity of metrology.

## 1. Introduction

The fourth-generation high-energy, low-emittance synchrotron source, HEPS (High Energy Photon Source), is currently in acceptance phase. The design parameter of the beam energy is up to 6 GeV, and the natural emittance reaches 34.2 pm rad (Jiao *et al.*, 2018 ▸). ID09 (the Hard X-ray Coherent Scattering beamline, HXCS) at HEPS is exclusively allocated to harness the source’s high brilliance and coherence through various techniques. ID09 at HEPS, which focuses on both coherent diffraction imaging (CDI) and X-ray photon correlation spectroscopy (XPCS), has multiple experiment requirements. The main experiment modes include ptychography, Bragg CDI (BCDI) and small-angle X-ray scattering (SAXS) XPCS. For ptychography and BCDI, the position of the sample is only 80 m. The distance from the end of the focal optical element, advanced KB mirrors (AKB-mirrors, consisting of a vertical focusing mirror, VFM, and a horizontal focusing mirror, HFM), to the sample is only 64 mm. As an important experiment mode, ptychography provides the foundation for obtaining non-destructive nanoscale reconstructions of the internal structure (Dong *et al.*, 2026 ▸; Zhang *et al.*, 2013 ▸). The ptychography setup requires a <10 nm high relative stability between the sample and the focus spot. However, the achievable resolution, which is highly correlated with position information, is ultimately limited by cumulative errors arising from instabilities and drifts, especially in complex 3D scanning systems (Dierolf *et al.*, 2010 ▸; Trtik *et al.*, 2013 ▸). Therefore, as one of the highest resolution displacement sensors, laser interferometers are essential for monitoring the relative position measurement, while, for BCDI, the stability requirement is not stringent. In the aspect of rotation movement, the ptychography setup requires 180° in yaw, while the BCDI setup requires 30° for pitch and roll, respectively. In addition, the sample environments used for both ptychography and BCDI include either a high vacuum (10^−3^ Pa) or ambient atmospheric conditions, and for AKB-mirrors UHV (10^−6^ Pa). To balance the stability of the ptychography setup and the rotation requirement of BCDI, the requirement of HV on the sample side must be fulfilled, whilst maintaining UHV for AKB-mirrors, and compact installation space. The ptychography setup and BCDI setup are separately designed and both installed in an HV vessel that is welded with a UHV AKB-mirrors vessel.

The interferometer metrology strategy is designed for a ptychography setup at HEPS ID09 under such limited conditions. Therefore, the measurement method should satisfy the following criteria. First, the sensors must be tiny, such as an Attocube 3010 interferometer or SmarAct Picoscale interferometer. A large-sized laser, such as a helium–neon laser (HeNe laser), is not suitable for separate vessels. Second, the metrology should have the ability to measure the relative vibration between the sample and the focal spot. Third, the sensor heads should be integrated in an independent metrology frame. Finally, the distance between the sensor head and the measured object should be as close as possible, or the Abbe error will affect the measurement results.

Several metrology strategies have been provided in ptychography setups or similar facilities. The I14 nanoprobe beamline at Diamond Light Source grapples with a parallel issue. A six-interferometer metrology strategy is implemented to maintain the beam and sample position with 3 nm stability. All the interferometers are mounted in the Kirkpatrick–Baez (KB) vacuum space and thereby track the pitch, roll, height and horizontal variations. The pixel size in the ptychographic reconstructions is 27 nm (Peach *et al.*, 2017 ▸; Quinn *et al.*, 2021 ▸). The compact metrology setup, which operates in two vessels under different vacuum levels, measuring the relative vibration between the sample and the focal spot, may have some inherent issues. First, the sensor heads do not appear to be mounted on an independent fixed frame, which may introduce additional reference bases, necessitate coordinate system conversions, or even incur vibration error. Moreover, the sensor heads arranged in both horizontal and vertical directions increase the occupied space and add mass inside the KB vessel, thereby introducing new challenges to system stability. Additionally, the sensor heads installed in the UHV KB vessel are relatively troublesome for subsequent maintenance. An instrument for 3D X-ray nano-imaging with a heterodyne HeNe laser frame at the cSAXS beamline (X12SA) of the Swiss Light Source provides differential position metrology. The interferometer signals are used by a proportional integral derivative (PID) controller to stabilize the piezo stage of the pinhole, maintaining its position to within 10 nm. A resolution of 18 nm in 2D imaging of a lithographic test pattern and 53 nm resolution in 3D test is achieved (Holler *et al.*, 2012 ▸), and the highly resolved quantitative X-ray ptychographic tomography of an extended object, yielding 16 nm isotropic 3D resolution, was presented later (Holler *et al.*, 2014 ▸). The optical element, a Fresnel zone plate (FZP), is very small and close to the sample, so the differential optical path can be easily implemented. However, the AKB-mirrors configuration typically spans about several hundred millimetres in length. The measurement optical path requires multiple transformations, making it considerably difficult to achieve. Besides, this large-sized laser is unsuitable for separate vessels. On the ptychographic nano-analytical microscope (PtyNaMi) on beamline P06 (PETRAIII), an interferometer measurement method is employed to monitor the sample position at high sampling frequencies. A ball lens retroreflector with half of the sphere coated with chromium is fixed under the sample. This setup has the potential for closed-loop position control (Schropp *et al.*, 2020 ▸). This method attaches the metrology frame to the optical elements’ mechanical structure to measure a relative vibration. But when the sample and optical elements are in two separate vessels, it is nearly impossible to implement. In Holler *et al.* (2014 ▸), an X-ray microscope featuring multilayer Laue lenses (MLLs) as nanofocusing optics employs nine fiber-optic interferometers to monitor the *X*, *Y* and *Z* positions of vertical, horizontal MLL and sample assemblies. The results of the ptychography measurement (13.3 nm in the horizontal plane and 32.9 nm in the vertical plane) exhibited the properties. A similar metrology strategy is shown in a scanning microscope at the 5-ID Submicron Resolution X-Ray Spectroscopy (SRX) beamline at National Synchrotron Light Source II (NSLS-II), but the KB mirror is applied to X-ray focusing (Nazaretski *et al.*, 2015 ▸). In TARUMA, the sub-microprobe station at CARNAUBA (Coherent X-Ray Nanoprobe Beamline) at Sirius, a complementary metrology frame with measured dynamics above 350 Hz was implemented. The capacitive probe is mounted on a circular frame and oriented toward the nominal sample position to uphold the Abbe principle. The KB granite is considered absolutely stable, so there is no provision for measuring the relative position between the sample and the focal spot (Nazaretski *et al.*, 2022 ▸). Besides, in the hard X-ray nanoprobe beamline BL13U at Shanghai Synchrotron Radiation Facility (SSRF) (Geraldes *et al.*, 2020 ▸), the hard X-ray nanoprobe at Taiwan Photon Source (TPS) (He *et al.*, 2024 ▸), a laser interferometric feedback system was integrated to maintain the displacement of the mirror or sample or both.

A fundamental metrology system is crucial for nano-level ptychography reconstruction, but the current setups have some common issues. First, the measurement scheme requires a large installation space, making it unsuitable for compact cross-vessel configurations. Second, the probe is not mounted on an independent frame, which introduces additional data conversion. Third, the scheme does not allow for relative measurements between the sample and the focal spot. Therefore, a beam-direction cross-vessel metrology method for ID09 is proposed, which fulfills the experiment requirements, addresses the issues in the current facilities, and satisfies the design criteria for metrology strategy. In the primary phase, it monitors and corrects the position between the focal spot and the sample to ensure stability. Furthermore, it provides reliable data support for ptychographic reconstruction. In Section 2, the metrology structure is described. The measurement algorithm is indicated in Section 3, and the measurement error is analyzed in Section 4. The experiments are demonstrated in Section 5, and conclusions given in Section 6.

## 2. Metrology structure

**Figure 1:**
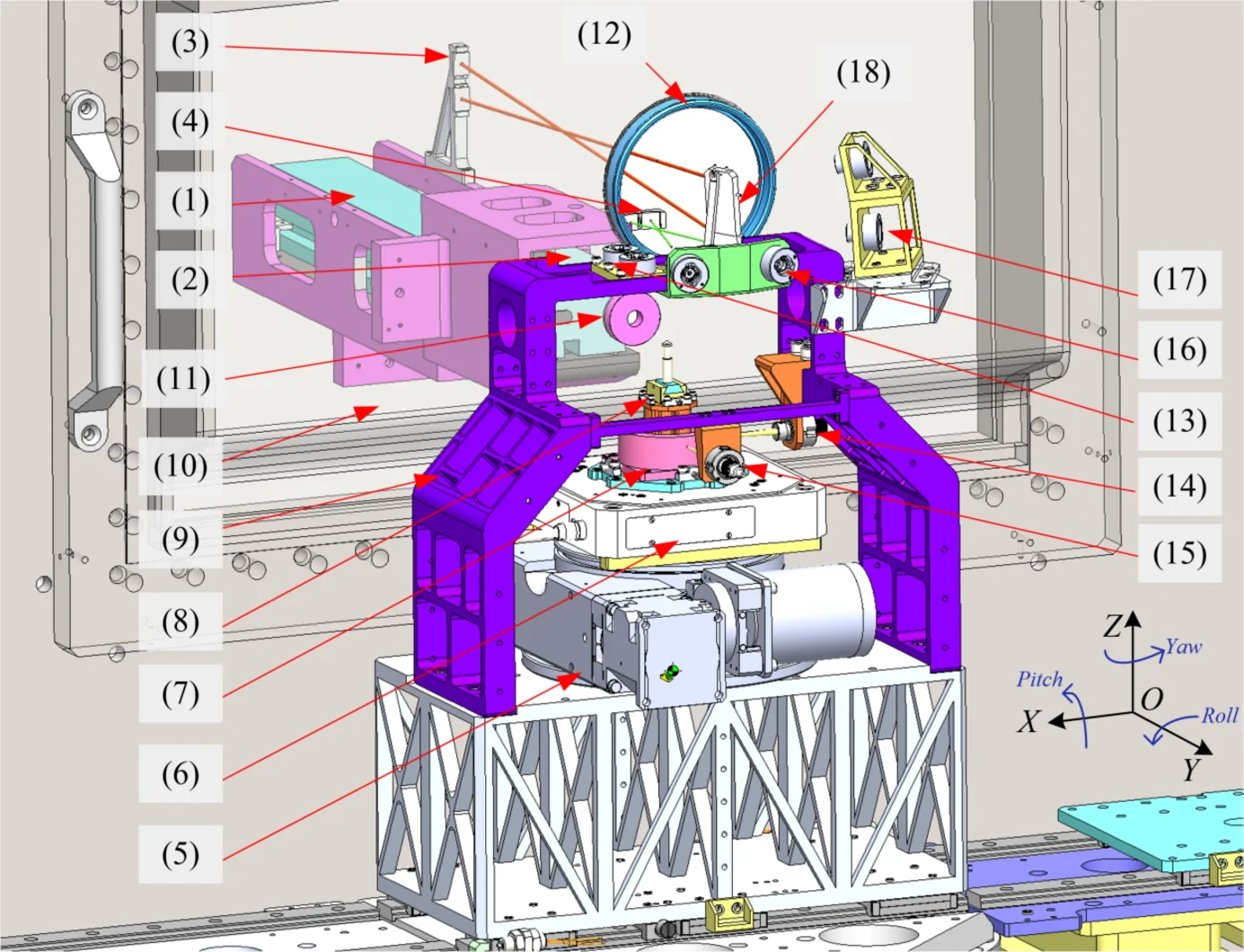
The metrology structure in the ptychography setup. (1) VFM. (2) HFM. (3) Bound-to-VFM plane mirrors. (4) Bound-to-HFM plane mirrors. (5) Rotation stage. (6) PI stage. (7) Cylinder retroflector. (8) Sample holder. (9) Invar metrology frame. (10) Chamber board. (11) Monocrystalline diamond window. (12) Sapphire window. (13) Three vertically oriented interferometers. (14) Primary focusing sensor head. (15) Secondary focusing sensor head. (16) HFM interferometer pair. (17) VFM interferometer pair. (18) Deflection plane mirror.

**Figure 2:**
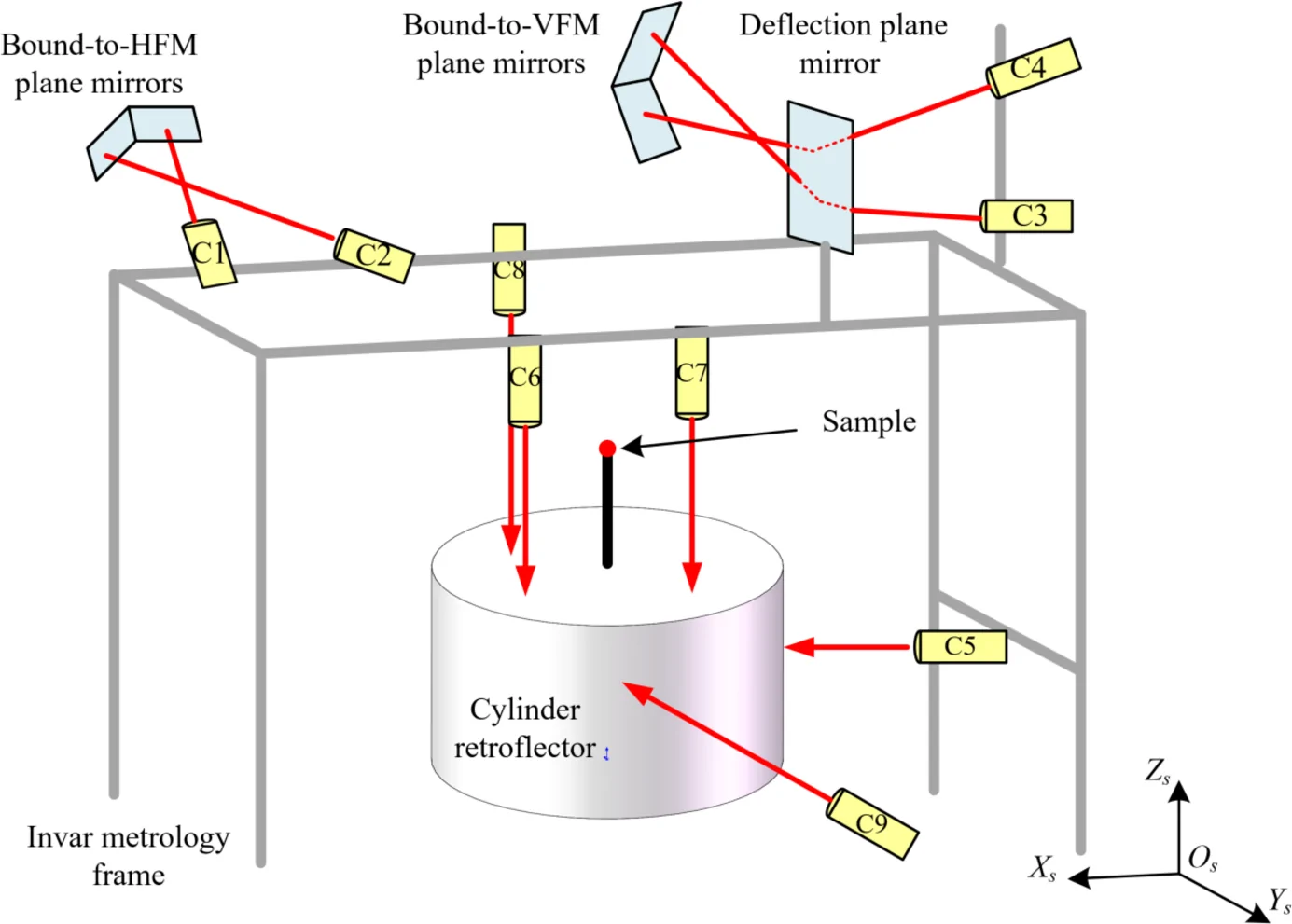
Metrology schematic diagram.

This metrology strategy is designed to monitor the relative position displacement between the sample and the focal spot, which is primarily influenced by the focusing optics, while also providing a foundation for ptychography reconstruction. Fig. 1 ▸ shows the metrology structure in the ptychography setup. A simplified metrology schematic diagram is shown in Fig. 2 ▸. The horizontal (*X*-axis) and vertical (*Z*-axis) directions are of primary interest. The beam direction is parallel to the *Y*-axis. The VFM (1), HFM (2), bound-to-VFM plane mirrors (3) and bound-to-HFM plane mirrors (4) are installed in the UHV vessel. The ptychography setup incorporates a rotation stage (5), stacked with a PI 3D nano piezoelectric displacement stage (6, PI stage, P-563), cylinder retroflector (7) and sample holder (8). The cylinder retroflector is made of polished aluminium with a gold coating. A total of nine interferometers (see also Fig. 2 ▸) are arranged on a dedicated, lightweight metrology Invar frame (9) in the HV vessel. The two vessels are separated by the chamber board (10). There are two windows on the chamber board, one is a monocrystalline diamond window (11) with 200 µm thickness and 10 mm effective aperture for X-rays, and the other is a sapphire (Al_2_O_3_) window (12) with 2 mm thickness and 90 mm aperture for lasers.

Three vertically oriented interferometers (13) serve to monitor the sample’s *Z*-axis displacement while simultaneously determining its roll and pitch for active correction. Two focusing sensor heads align to the center of the cylindrical reflecting surface. The primary focusing sensor head (14) monitors the *X*-axis, and the secondary focusing sensor head (15) determines the *Y*-axis displacement. The *X*-axis and *Z*-axis movements of HFM and VFM are measured by two pairs of interferometers. The HFM interferometer pair (16) is configured at a 26° angle, while the VFM interferometer pair (17) is oriented at an 8° angle. For accuracy reasons, the angle between two sensor heads should be as large as possible. However, it is limited by the installation space, the sensor heads’ layout of approximate beam direction, and the effective aperture of the sapphire window. Although these two sensor heads’ design for one-dimensional monitoring might be perceived as redundant, this design makes sure that all the sensor heads can concentrate on an independent metrology frame, which can be designed very compactly for switching the experimental modes easily. Besides, the two sensor heads can be computed as a difference operator to compensate for the drift in the other direction. In practice, it was observed that there are two reflector laser points for one sensor head in VFM or HFM interferometer pairs. One is from the bound-to-VFM or bound-to-HFM plane mirrors, and another is from the sapphire window. In the VFM interferometer pairs, the angle is very small, and the optical path is not long enough to distinguish these two laser points. Hence, we added a deflection plane mirror (18) extending the optical path while ensuring a more compact structure.

## 3. Metrology algorithm

**Figure 3:**
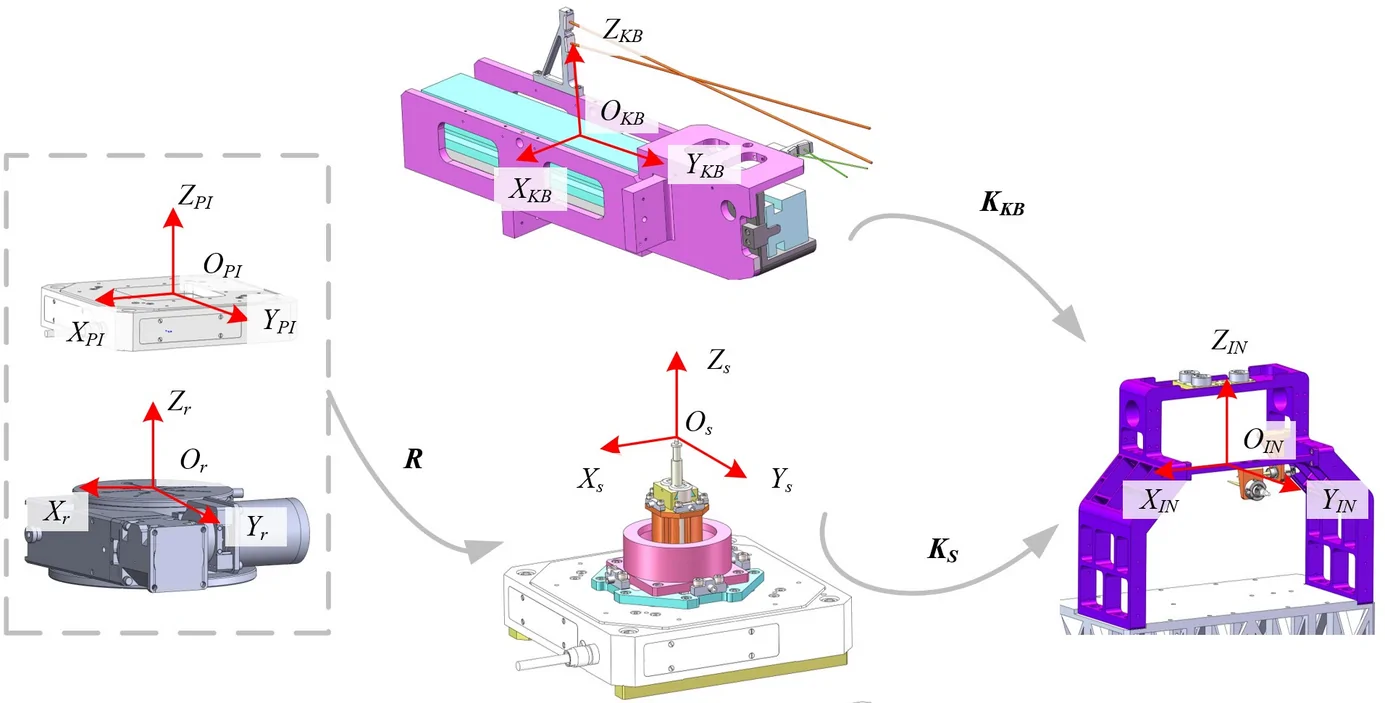
Coordinate systems translations.

The method proposed here is more general because it extends the measurement to include the *Y*-axis and applies to a wider range of similar equipment, even though the ptychography setup in ID09 primarily targets drift in the *X* and *Z* directions. Fig. 3 ▸ shows the coordinate transformation relationship. There are five coordinate systems to note: the AKB coordinate system *O*_KB_–*X*_KB_*Y*_KB_*Z*_KB_, the sample coordinate system *O*_S_–*X*_S_*Y*_S_*Z*_S_, the rotation coordinate system *O*_r_–*X*_r_*Y*_r_*Z*_r_, the PI stage coordinate system *O*_PI_–*X*_PI_*Y*_PI_*Z*_PI_ and the interferometer frame coordinate system *O*_IN_–*X*_IN_*Y*_IN_*Z*_IN_. The AKB and sample coordinate systems are bridged by the interferometer frame coordinate system. The rotation coordinate system and PI stage coordinate system are related to the sample coordinate system. Any relative movement, including translation and rotation, introduces changes in the interferometer readings, as modeled by equation (1). The first three rolls in equation (1) express the *Z*, *Y*, *X* translations, the fourth and fifth rolls express the roll and pitch attitude. *C*_*i*_ (*i* = 1,…, 9) represents the interferometer value of the corresponding serial numbers of different sensor heads in Fig. 2 ▸. *Z*_S_, *Y*_S_ and *X*_S_ are the sample positions. These values can likewise be acquired from the capacitive sensor embedded in the commercial PI stage to calibrate its linear calibration. Parameters *k*_*ij*_ (*i* = *Z*, *Y*, *X*, *R*, *P*; *j* = *z*, *y*, *x*) are the coefficients between the *i* dimension (including *Z*-axis, *Y*-axis, *X*-axis, roll and pitch) in the interferometer frame coordinate system and the *j*-axis direction in the sample coordinate system. The linear calibration of *k*_*ij*_ is performed by moving the PI stage along one direction at a time while locking the others. This allows equation (1) to be dimensionally reduced to equation (2) for parameter identification. In this case, [*Z*_S_, *Y*_S_, *X*_S_] is equal to [*Z*_PI_, *Y*_PI_, *X*_PI_], and equation (1) can be rewritten as equation (3),
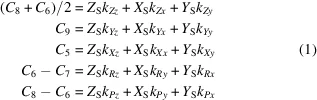

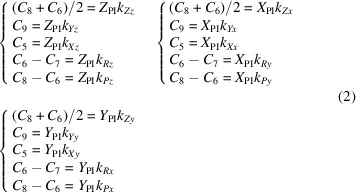

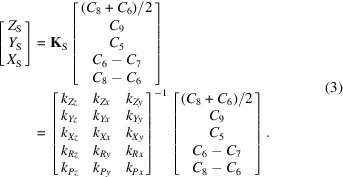
When the rotation stage works, the rotation factor ***R*** is introduced with [*Z*_PI_, *Y*_PI_, *X*_PI_] to determine the sample position [*Z*_S_, *Y*_S_, *X*_S_] for calibration, resulting in the augmented model given by equation (4), where α denotes the rotation angle,
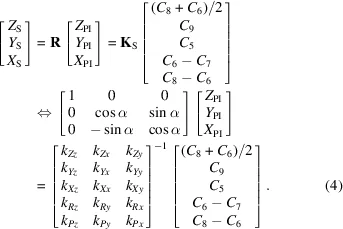
The interferometer pairs *C*_1_/*C*_2_ and *C*_3_/*C*_4_ are assigned to measure *X*-axis disturbances on the HFM and *Z*-axis disturbances on the VFM, respectively. If a disturbance in the *Y*-axis is necessary, *C*_1_/*C*_2_ or *C*_3_/*C*_4_ can also be used to measure. The interferometer values and AKB-mirrors configuration’s disturbance *Z*_KB_, *Y*_KB_, *X*_KB_, satisfy a linear relationship as shown in equation (5). Parameters *k*_KB*z*_, *k*_KB*y*_, *k*_KB*x*_ are the slope coefficients, which can also be calibrated by AKB-mirrors position adjustment mechanically. Besides, the position of the AKB-mirrors attitude adjustment mechanism and the sample stage is defined by the laser tracker in advance. To ensure the relative position between the AKB-mirrors and the sample, the relative position errors *Z*_e_, *Y*_e_ and *X*_e_ are defined in equation (6). This value can correct the imaging quality or motor feedback,
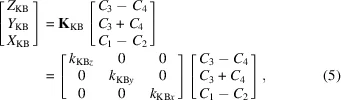

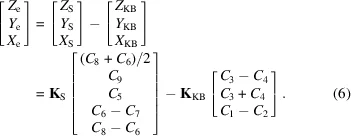
In our application, only the disturbance in the *X*- and *Z*-axes needs to be focused on. Hence, the transform between the PI stage and the interferometer frame coordinate system in equation (3) can be simplified as equation (7). After rotation, *Z*_S_ and *X*_S_ for calibration are shown in equation (8). The *Z*-axis and *X*-axis two-dimensional disturbance in the AKB-mirrors configuration are expressed in equation (9). The relative position error *Z*_e_, *X*_e_ is defined in equation (10).
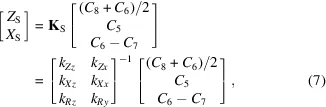

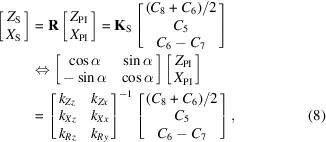

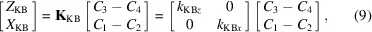

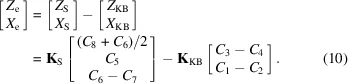
Because the angle between *C*_3_ and *C*_4_ is set to 8°, and that between *C*_1_ and *C*_2_ is configured as 26°, the theoretical values of *k*_KB*z*_ and *k*_KB*x*_ can be calculated as equation (11), where *θ*_*Z*_ and *θ*_*X*_ are 4° and 13°, respectively. In equation (8), parameters *k*_*Zz*_ and *k*_*Xx*_ account for the vast majority of the proportion; their absolute values should be close to 1, and other values in **K**_s_ approach zero,
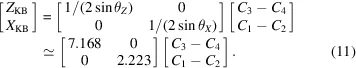


## 4. Error analysis

This section presents a detailed analysis quantifying key error sources in the measurement, addressing errors arising from the Abbe effect, installation misalignment, surface figure deviations, and other sources.

### 4.1. Asymmetric installation

**Figure 4:**
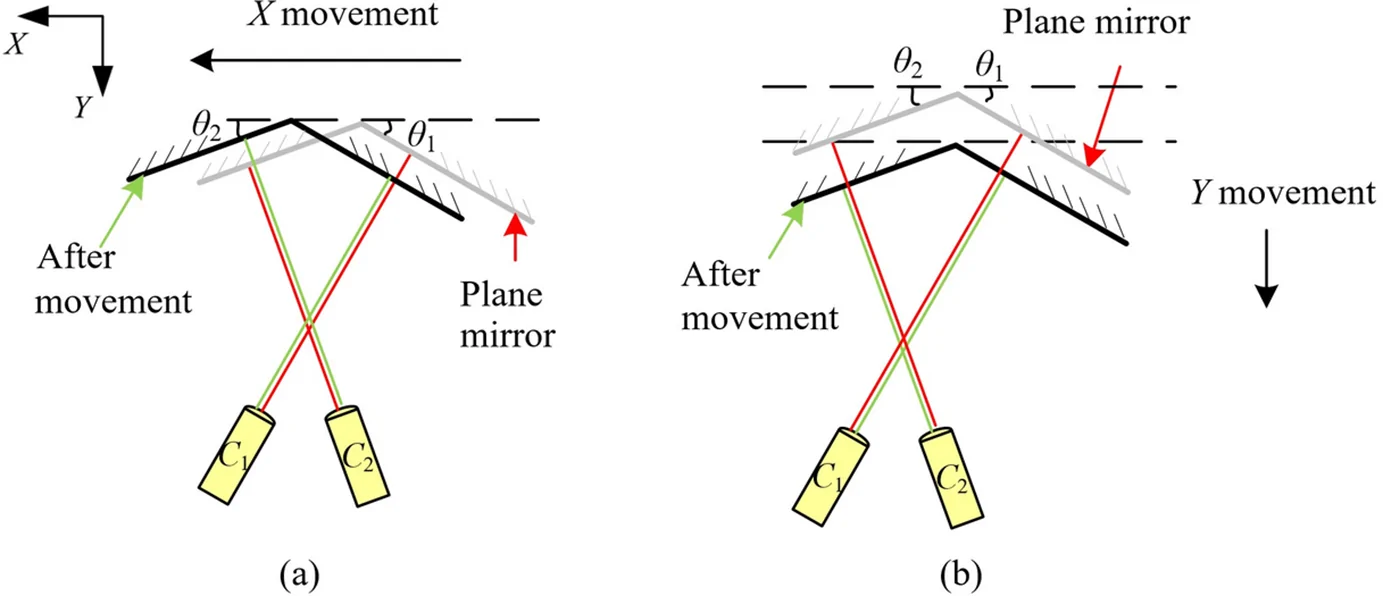
Error caused by the asymmetric installation of *C*_1_ and *C*_2_. The movement of the mirror along the *X*-axis (*a*) and *Y*-axis (*b*) both causes a variation in the sensor heads’ reading.

For the AKB-mirrors position measurement, the interferometer pair *C*_1_/*C*_2_ of HFM is described as an example in Fig. 4 ▸. The asymmetric installation of *C*_1_ and *C*_2_ leads to *X*_KB_ not being calculated as equation (11) ideally. *X*-axis and *Y*-axis movement both affect the interferometers’ reading. *X*_KB_ is recalculated using equation (12), 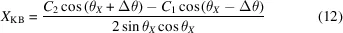
where
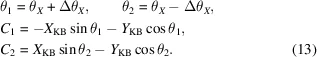
Under the assumption that *C*_1_ = *C*_2_ = 100 nm, θ_*X*_ = 13° and Δθ_*X*_ = 1°, the error of Δ*X*_KB_ computed as equation (14) is about 1.80 nm. Other things being equal, θ_*Z*_ is 4° for VFM; the derivation of the error of *Z*_KB_ is similar, and the value is 1.75 nm. If *C*_1_ and *C*_2_ are not equal, this value would be larger. However, this inescapable systemic error caused by asymmetric installation can be eliminated by calibrating and adjusting the parameter **K**_KB_, as the error is almost linear,
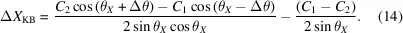


### 4.2. Abbe error

**Figure 5:**
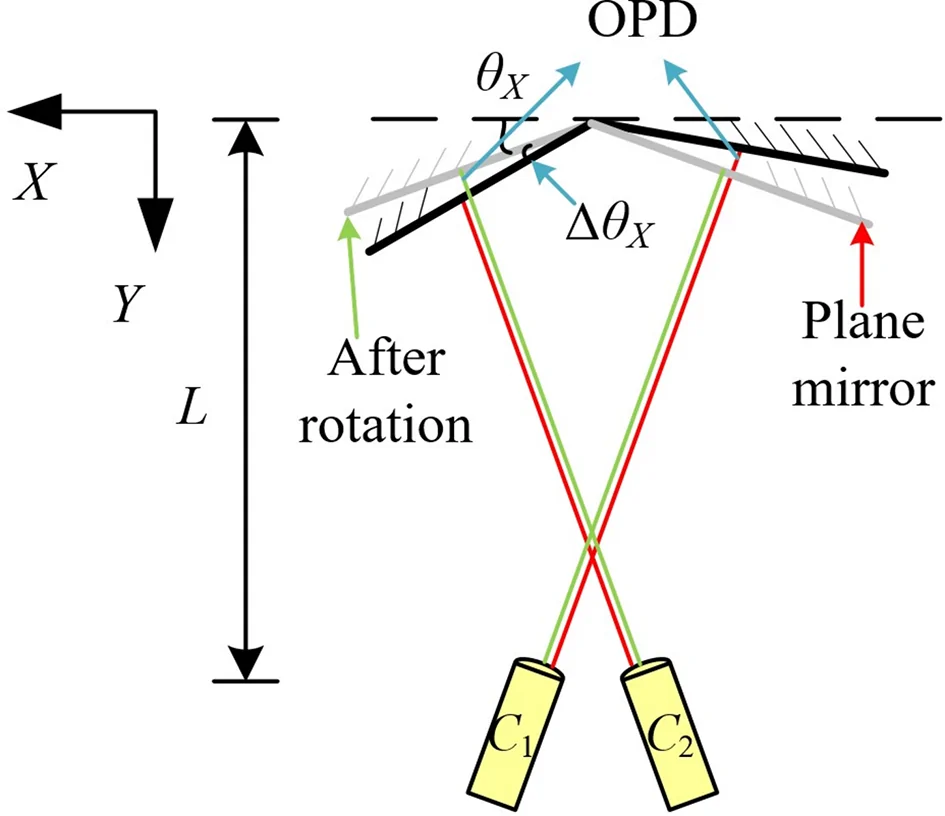
Abbe error analysis in the AKB-mirror configuration.

Another dominant contribution to measurement uncertainty is the Abbe error. As shown in Fig. 5 ▸, the rotation of the mirrors produces an optical path difference (OPD), resulting in measurement error. The Abbe error is controlled by static and compact mechanical design and environmental stability, and the error Δ*X*_KB_ is computed as equation (15), where *L* is the measurement arm between the bound-to-HFM plane mirrors and sensor heads. Δθ_*X*_ here is the relative angle stability between the AKB-mirrors configuration and the sensor head,
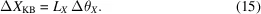
For example, if Δθ_*X*_ is less than 10 nrad and the *L*_*X*_ of HFM is less than 150 mm, the Abbe error is kept below 1.50 nm for the HFM. For VFM, the measurement arm *L*_*Z*_ is less than 400 mm, and the Abbe error is assumed to be less than 4.00 nm. This Abbe error can only be minimized by enhancing the system stability or shortening the measurement arm.

### 4.3. Mirrors surface error

Machining surface errors of bound-to-HFM or bound-to-VFM can be classified into two categories: slope error and residual height error. The residual height error is much less than the 30 nm requirement of the plane mirror surface roughness, because the vibration range between the AKB-mirror configuration and sensor heads is minimal. This error is able to be calibrated or even ignored. The slope error mainly affects **K**_KB_. As shown in equations (9) and (11), Δ**K**_KB_, which expresses the variation values of parameter **K**_KB_, is computed as equation (16). If Δθ is assumed to be 1 µrad, the absolute values of Δ*k*_KB*z*_ and Δ*k*_KB*x*_ are less than 5 × 10^−4^. Usually, the instability of the AKB-mirror configuration is less than 100 nm, and a 1 µrad surface error is achievable. Hence, the influence of slope error is less than 0.05 nm and can also be ignored,
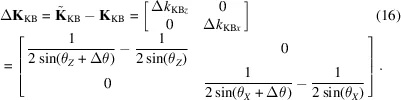


### 4.4. Vibration impact of the environment-isolating sapphire window

**Figure 6:**
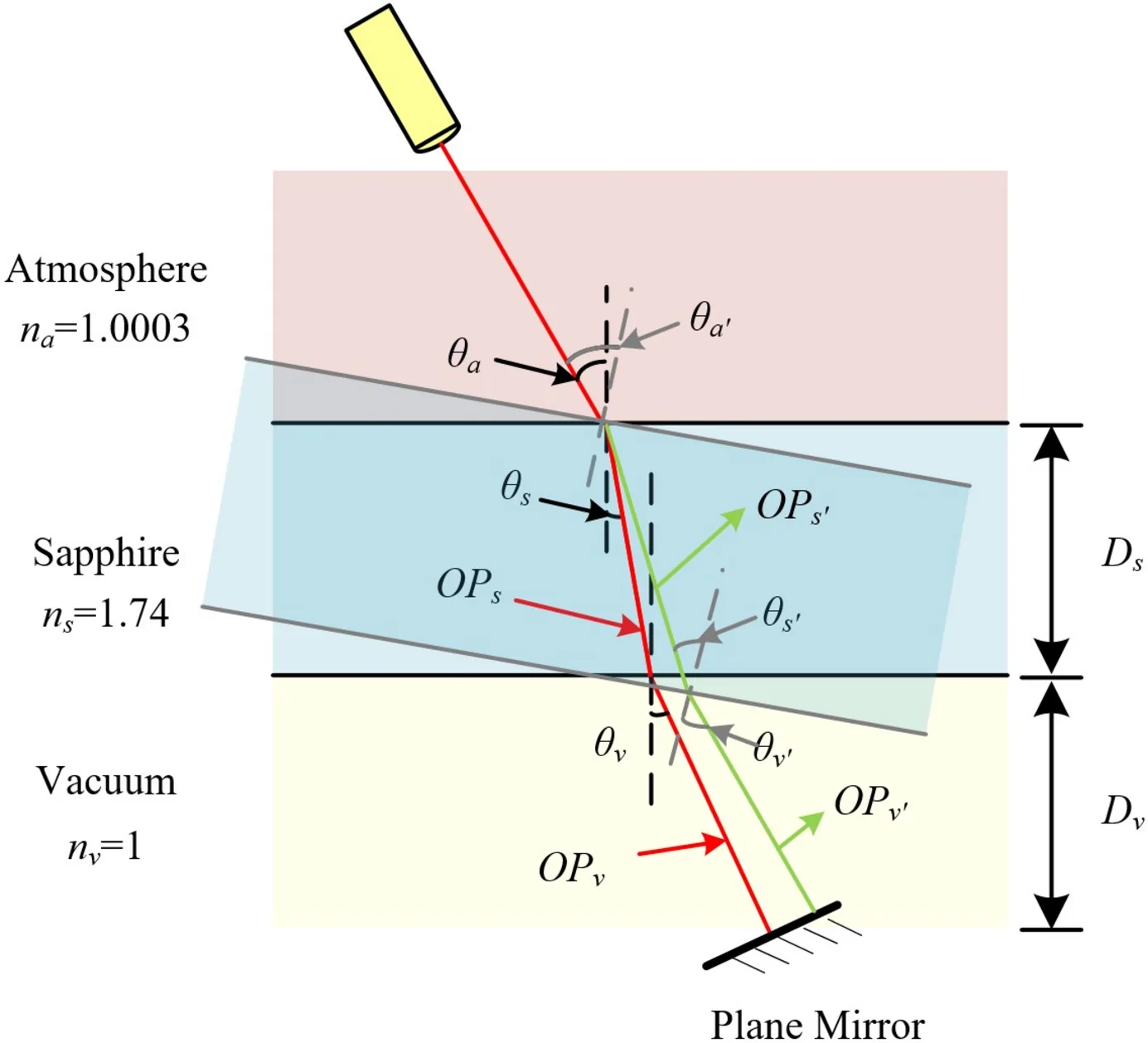
The rotation variation of the sapphire window affects the measurement accuracy.

Although the inhomogeneity of the sapphire window is out of consideration, the vibration of the sapphire window and different vacuum environments also affect the measurement accuracy. As shown in Fig. 6 ▸, the optical path passes through three different media with varying refractive indices: atmosphere, sapphire and vacuum. Usually, the optical path is along the red line. The total optical paths are expressed as the sum of optical paths in sapphire OP_s_ and in vacuum OP_v_. A rotation variation Δθ caused by vibration makes the optical path along the green line. The total optical paths become the sum of optical paths OP_s′_ and OP_v′_. The OPD, which is also the measurement error, is computed as equation (17),
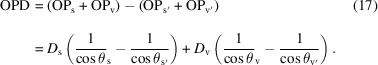
According to Snell’s law, angle parameters θ_s_, θ_v_, θ_s′_ and θ_v′_ are given by equation (18),
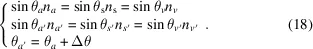
In our application, the refractive indices of the atmosphere *n*_a_, sapphire *n*_s_ and vacuum *n*_v_ are 1.0003, 1.74 and 1, respectively. The thickness of the sapphire window *D*_s_ is 2 mm. *D*_v_ is considered equal to the measurement arm. Δθ is assumed to be 100 nrad. Angle θ_a_ for HFM and VFM is thought to be θ_*X*_ and θ_*Z*_. Under the above conditions, the OPD for HFM is calculated as 3.57 nm and for VFM is 2.81 nm. Besides, the error introduced by the translational vibration is related to the refractive indices in the atmosphere and vacuum, which can be approximated as three ten-thousandths of the vibration amplitude.

In brief, for the AKB-mirrors position measurement, the parameter configuration is given in Table 1 ▸. The quantitative error analysis is given in Table 2 ▸.

### 4.5. Errors in sample position measurement

**Figure 7:**
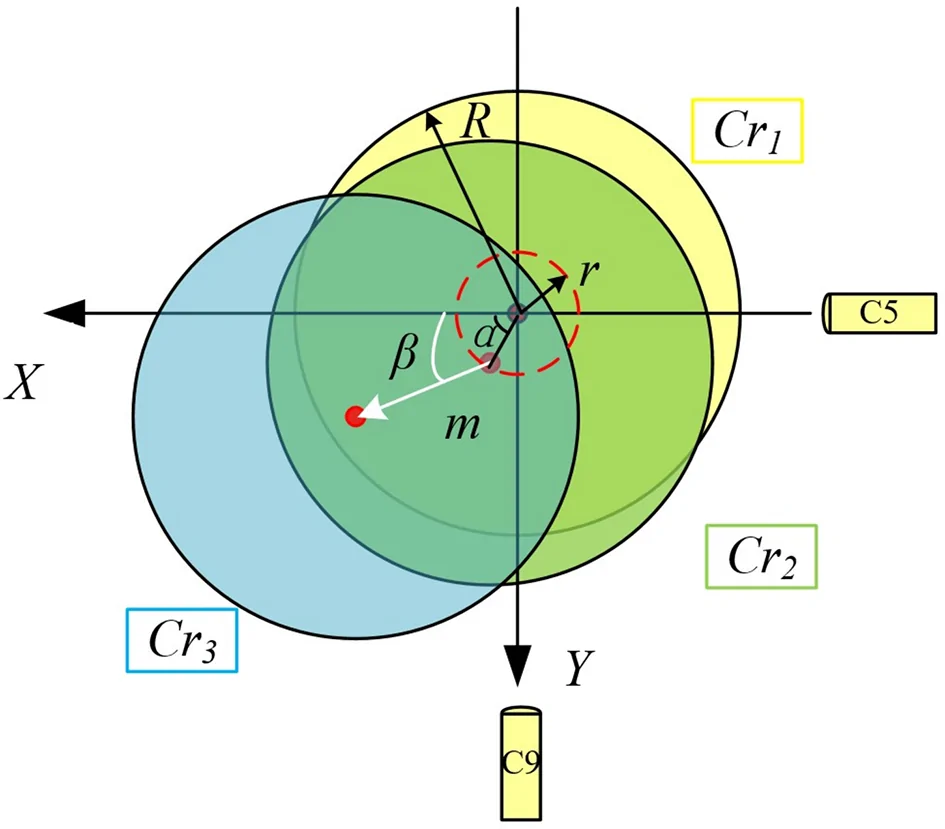
A top view of the cylinder retroflector to explain the error in the sample position measurement. Cr_1_ (yellow circle) is the ideal position of the cylindrical retroflector, whose radius is parameter *R*. Cr_2_ (green circle) is the real position. For the installation error, the distance between Cr_1_ and Cr_2_ is expressed as parameter *r*. Parameter α denotes the angle formed by the *X*-axis and the line joining the center of Cr_1_ and Cr_2_. Parameter β is defined as the angle from the movement direction of Cr_2_ (white arrow line) and the *X*-axis. *C*5 and *C*9 are the interferometer’s sensor head, see also in Fig. 2 ▸.

**Figure 8:**
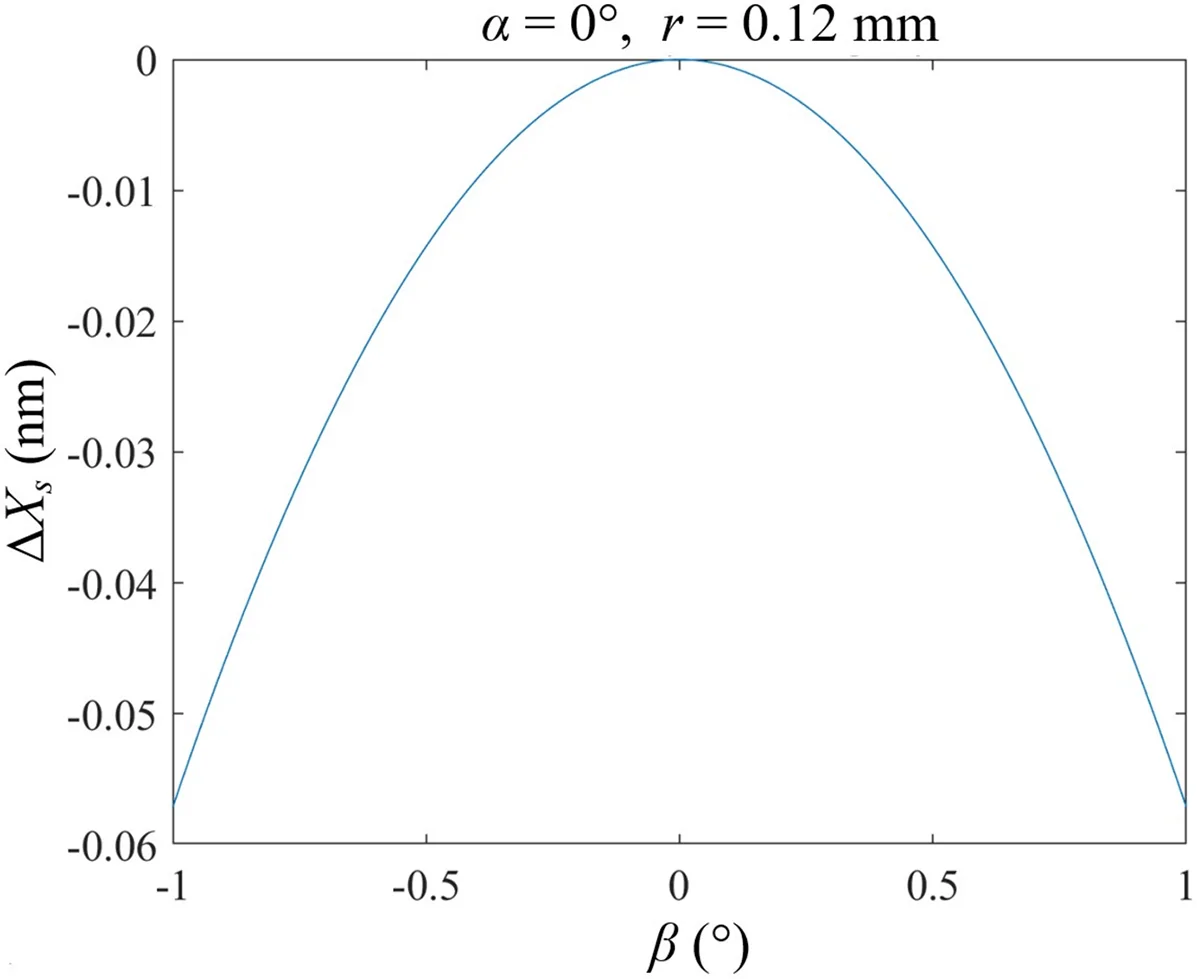
When only 2D scanning is operated, the Δ*X*_s_ varies with the angle β between the movement direction and the *X* axis. Parameter α is set to 0°, and *r* is set to 0.12 mm.

In Fig. 2 ▸, there are five sensor heads for the sample position monitor. The three sensor heads *C*_6_, *C*_7_ and *C*_8_, positioned above the sample, measure the pitch and roll of the sample stage, enabling compensation of the Abbe error. However, an additional significant error source arises from the misalignment between the rotational stage center and the cylindrical retroflector. The top view of the cylinder retroflector is shown in Fig. 7 ▸. The sensor heads *C*_5_ and *C*_9_ are positioned along the *X* and *Y* axes. The yellow circle Cr_1_ expresses the ideal position of the cylindrical retroflector, whose center coincides with the origin of the rotation coordinate system. The real position is shown as the green circle Cr_2_, rotating around the center of the rotation stage with radius *r*. Even in this case, there is no extra error when the movement direction of the translation stage is exactly along the *X* or *Y* axis. Parameter β is the angle between the movement direction (white arrow line) and the *X*-axis. The rotation angle α and the curvature of the cylinder introduce extra measurement error. The equations of Cr_2_ and Cr_3_ are shown in equation (19). Parameter *R* is the radius of the cylinder retroflector, and *m* expresses the half maximum movement range. To get the interferometer value on the *X*-axis, *Y*_s_ is set to zero. *X*_s_ is calculated using equation (20). Assuming that the influence of the cylinder retroflector’s curved surface is not considered, the measurement value 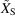 of *C*_5_ in the *X*-axis should be shown in equation (21). The sensor measurement error Δ*X*_s_ is expressed in equation (22).
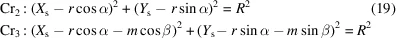

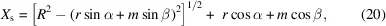

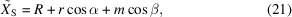

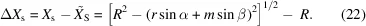
In our application, when only 2D scanning is operated, the angle α can be set to 0°. The radius of the cylinder retroflector *R* is 60 mm, and the half-maximum movement range of the PI stage *m* is 0.15 mm. Rotation radius *r* is related to the installation accuracy and is set to 0.12 mm. Parameter β is set in the range [−1, 1]°. Δ*X*_s_ varies with angle β as shown in Fig. 8 ▸. The results indicate that the error caused by misalignment, the curved surface of the cylindrical retroflector, and the movement angle are negligible during 2D scanning operations.

**Figure 9:**
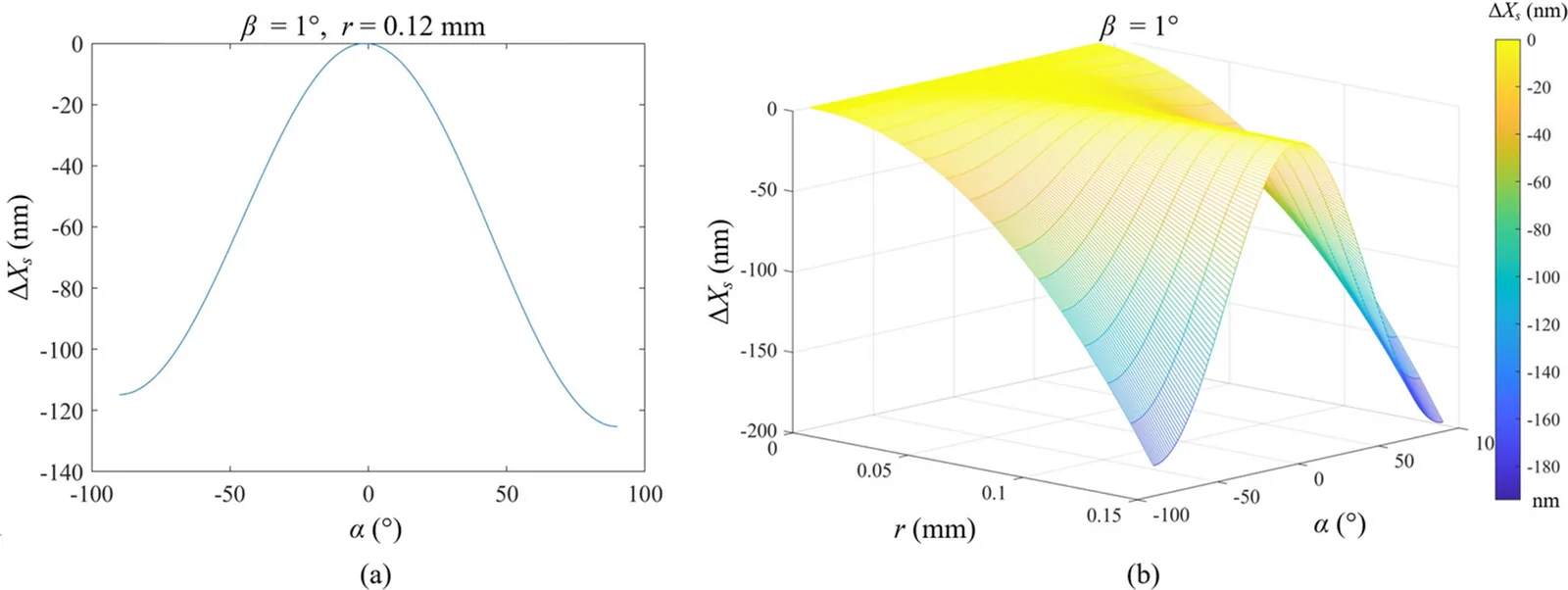
(*a*) When 3D scanning is operated, Δ*X*_s_ varies with the angle α between the movement direction and the *X* axis. Parameter β is set to 1°, and *r* is set to 0.12 mm. (*b*) *X*_s_ varies with the angle α and *r*. The range of α is [−90, 90]°, and of *r* is [0, 0.15] mm.

For 3D scanning operations where β and *r* are constant (*e.g.* 1° and 0.12 mm), the resulting error in Δ*X*_S_ [Fig. 9 ▸(*a*)] reaches ∼120 nm over a ±90° rotation of α, based on equation (22). Consequently, the parameter **K**_S_ is not angle-invariant and must be corrected. Fig. 9 ▸(*b*) shows the relationship of Δ*X*_S_, *r* and α. Table 3 ▸ presents the detailed data as specified in Fig. 9 ▸(*b*). To balance calibration complexity with precision, *r* is recommended to be less than 50 µm. With a 15° interval for the **K**_S_ correction, the error is limited to about 2 nm. Actually, *r* depends on the installation accuracy. To ensure a ∼2 nm error or less, the interval for the **K**_S_ correction should be adjusted according to different *r*.

## 5. Test and experiments

**Figure 10:**
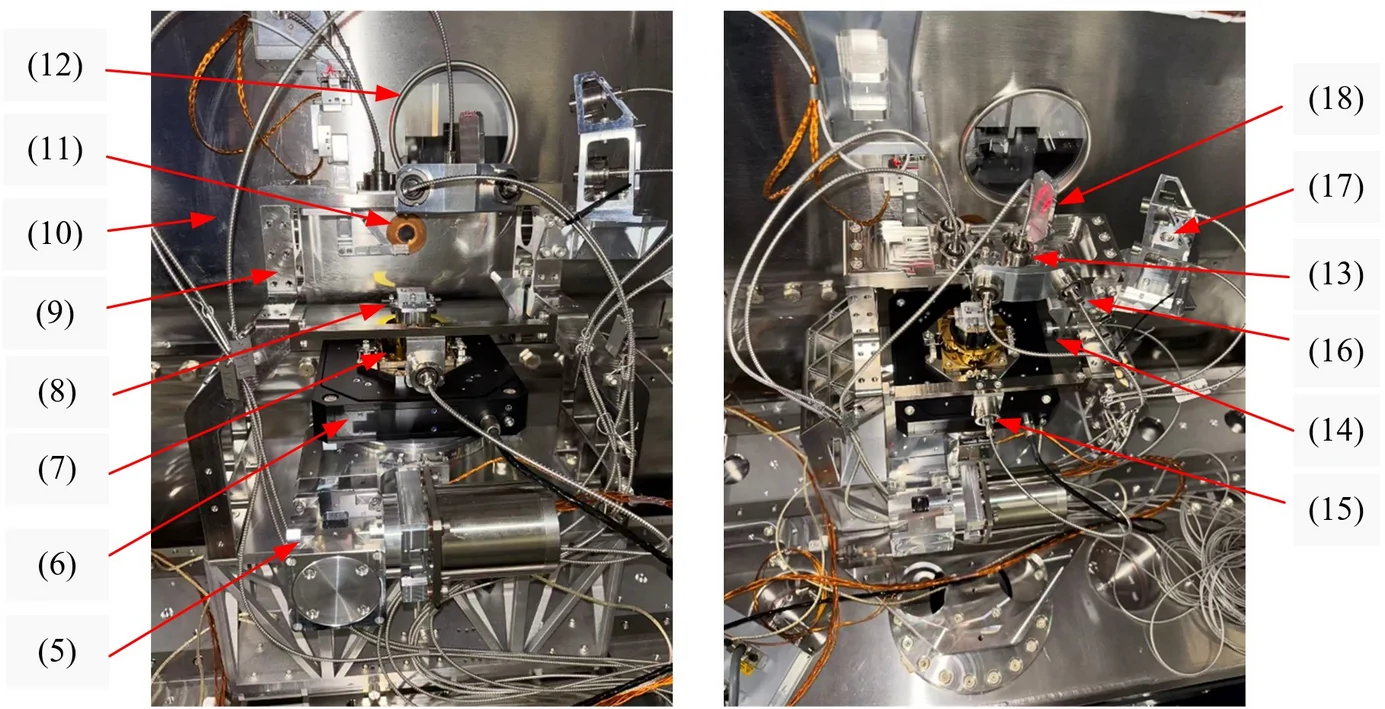
The interferometer strategy in the ID09 ptychography setup.

**Figure 11:**
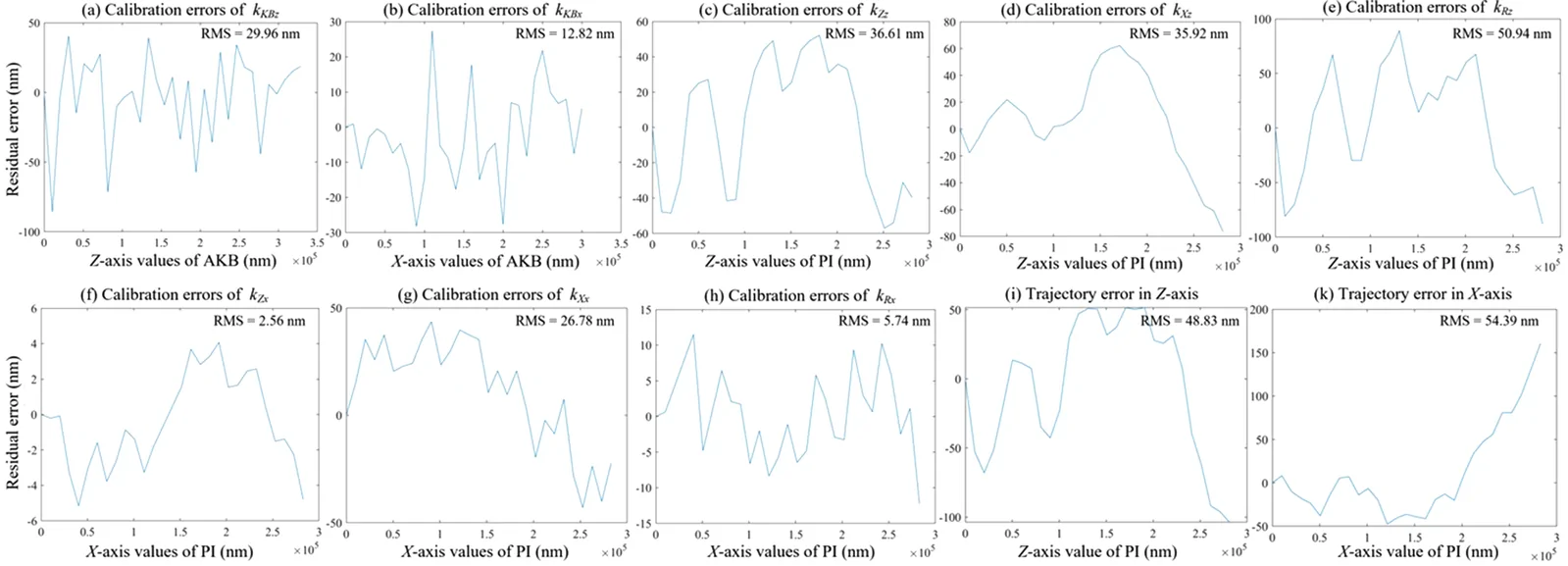
The calibration error distribution.

**Figure 12:**
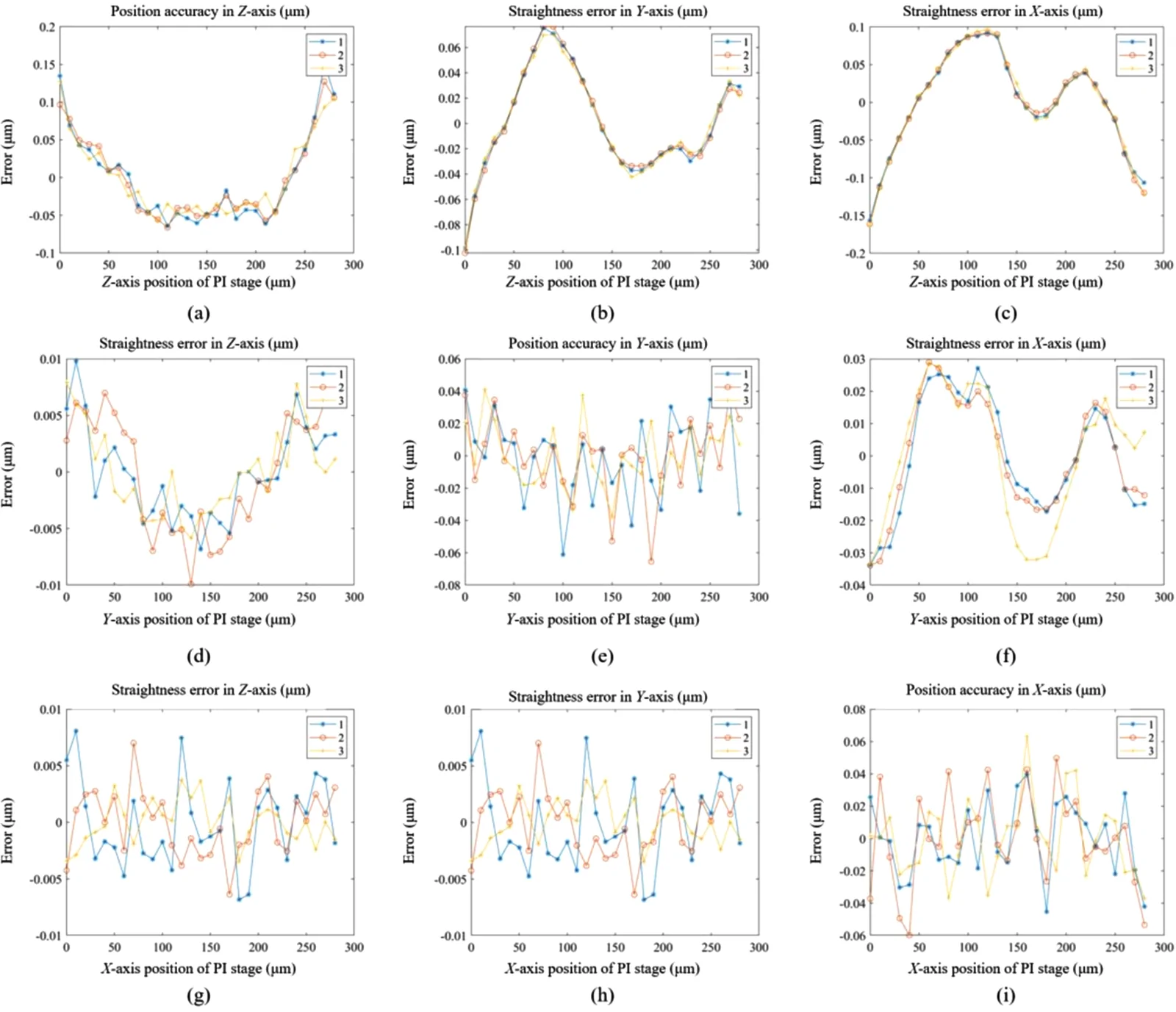
The difference between interferometers and the PI stage values after a linear fit. Panels (*a*), (*b*) and (*c*) present the measurement results along the *Z*-, *Y*- and *X*-axes, respectively, as the PI stage moves through its *Z*-axis stroke. Similarly, panels (*d*), (*e*) and (*f*) show the results along the *Z*-, *Y*- and *X*-axes when the PI stage moves through its *Y*-axis stroke. Panels (*g*), (*h*) and (*i*) show the *Z*-, *Y*- and *X*-axes results when it moves through its *X*-axis stroke.

The interferometer strategy in the ID09 ptychograpy experiment setup is illustrated in Fig. 10 ▸. The numbers in Fig. 10 ▸ correspond to those in Fig. 1 ▸. The calibrated parameters of **K**_KB_ and **K**_s_ are shown in equation (25) when the rotation angle α is equal to 0°. The matrix **K**_KB_ is calibrated by the movement mechanism, and the matrix **K**_S_ is calibrated by the PI stage using the method described in Section 3, shown in equations (2) and (9). The values in equation (11) correspond to the ideal theoretical scenario, where the angle between *C*3 and *C*4 is precisely 8° and that between *C*1 and *C*2 is precisely 26°. Given that asymmetric installation (see Section 4.1) is unavoidable, the actual results in equation (25) may deviate from these theoretical values, either larger or smaller. Nevertheless, they are expected to remain close to the theoretical predictions. The PI stage used for linear calibration has a stroke range of −140 µm to 140 µm. The readout of the PI stage and the interferometers are acquired every 10 µm. First, the parameters *k*_*Zz*_, *k*_*Xz*_, *k*_*Rz*_, *k*_*Zx*_, *k*_*Xx*_ and *k*_*Rx*_ are linearly calibrated via linear regression, corresponding to the respective rows in equation (2). For instance, *k*_*Zz*_, located in the first row, first term in equation (2), can be computed using equation (23). The error *E*_*kZz*_ in each point is given by equation (24), where *i* is from 1 to *N*, and *N* = 29 is the total number of calibration points. For other parameters, the algorithm is similar, and the errors are expressed as *E*_*kXz*_, *E*_*kRz*_, *E*_*kZx*_, *E*_*kXx*_, *E*_*kRx*_. When α is equal to zero, the calibration error RMS of *E*_*kZz*_, *E*_*kXz*_, *E*_*kRz*_, *E*_*kZx*_, *E*_*kXx*_ and *E*_*kRx*_ are 36.61 nm, 35.92 nm, 50.94 nm, 2.56 nm, 26.78 nm and 5.74 nm, respectively. The calibration RMS of *E*_*k*KB*z*_ and *E*_*k*KB*x*_ are 29.96 nm and 12.82 nm, respectively. Furthermore, **K**_S_ are verified by an additional linear trajectory, where the *X* and *Z* axes of the PI stage move in a synchronized motion. The residual errors RMS in the *Z* and *X* axes are 48.83 nm and 54.39 nm, respectively. All these results are summarized in Fig. 11 ▸. 
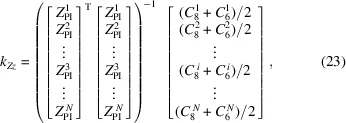

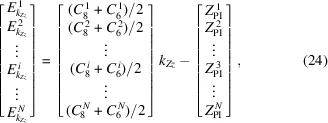

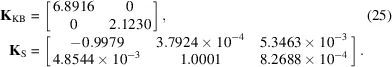
To illustrate the reason for the relatively large calibration error of **K**_S_ in Fig. 11 ▸, the performance of the PI stage (P-563) is measured separately. The interferometer optical path is simplified as three probes in three axes. The PI stage travels sequentially along the *X*-, *Y*- and *Z*-axes with a stroke of 280 µm, performing three motions per axis. Data from the PI stage and the interferometer are acquired every 10 µm. Regardless of the direction of motion, all three interferometers acquire data. Compared with conventional evaluation, when the PI stage moves along an axis, the position accuracy in this direction is given and the straightness errors in the other two orthogonal axes are also provided. In addition, the repeatabilities in three directions are also calculated. The results are shown in Fig. 12 ▸ and the quantitative values are given in Table 4 ▸. The nine-grid diagram corresponds one-to-one with the nine-grid results in the table. For example, when the PI stage moves through its *Z*-axis stroke, the translation errors in three directions (*Z*-axis, *Y*-axis, *X*-axis) are recorded in Figs. 12 ▸(*a*), 12(*b*), and 12(*c*), respectively. The error along its movement direction (*Z*-axis) denotes position accuracy, and the errors in the two orthogonal axes (*Y*-axis and *X*-axis) demonstrate the straightness errors. Similarly, Figs. 12 ▸(*d*), 12 ▸(*e*) and 12(*f*) show the results along the *Z*-, *Y*- and *X*-axes when the PI stage moves through its *Y*-axis stroke. So Fig. 12 ▸(*e*) expresses the position accuracy in the *Y*-axis, and Figs. 12(*d*) and 12(*f*) show the straightness errors. Figs. 12 ▸(*g*), 12 ▸(*h*) and 12(*i*) show the results along the *Z*-, *Y*- and *X*-axes when the PI stage moves through its *X*-axis stroke. And Fig. 12 ▸(*i*) expresses the position accuracy in the *X*-axis, and Figs. 12(*g*) and 12(*h*) show the straightness errors. The position accuracies in the *Z*-, *Y*- and *X*-axes are 55.80 nm, 23.17 nm and 24.94 nm, respectively. The repeatabilities of the *Z*-, *Y*- and *X*-axes are 9.48 nm, 16.04 nm and 18.82 nm, respectively. Despite high repeatability, the absolute positioning accuracy is compromised. Two possible reasons may account for this. First, in measurement, though the linear error has been calibrated, the Abbe error may still exist. As shown by Tang *et al.* (2013 ▸), even considerable errors can arise when two nominally identical instruments are used to measure against each other, let alone when the instruments are of different types and the measurement distances vary. Second, the mechanism of the PI stage is a black box to us. The internal mechanism itself may have nonlinearities. Though sub-hundred-nanometre accuracy may not be extremely precise, it is sufficient for linear calibration.

**Figure 13:**
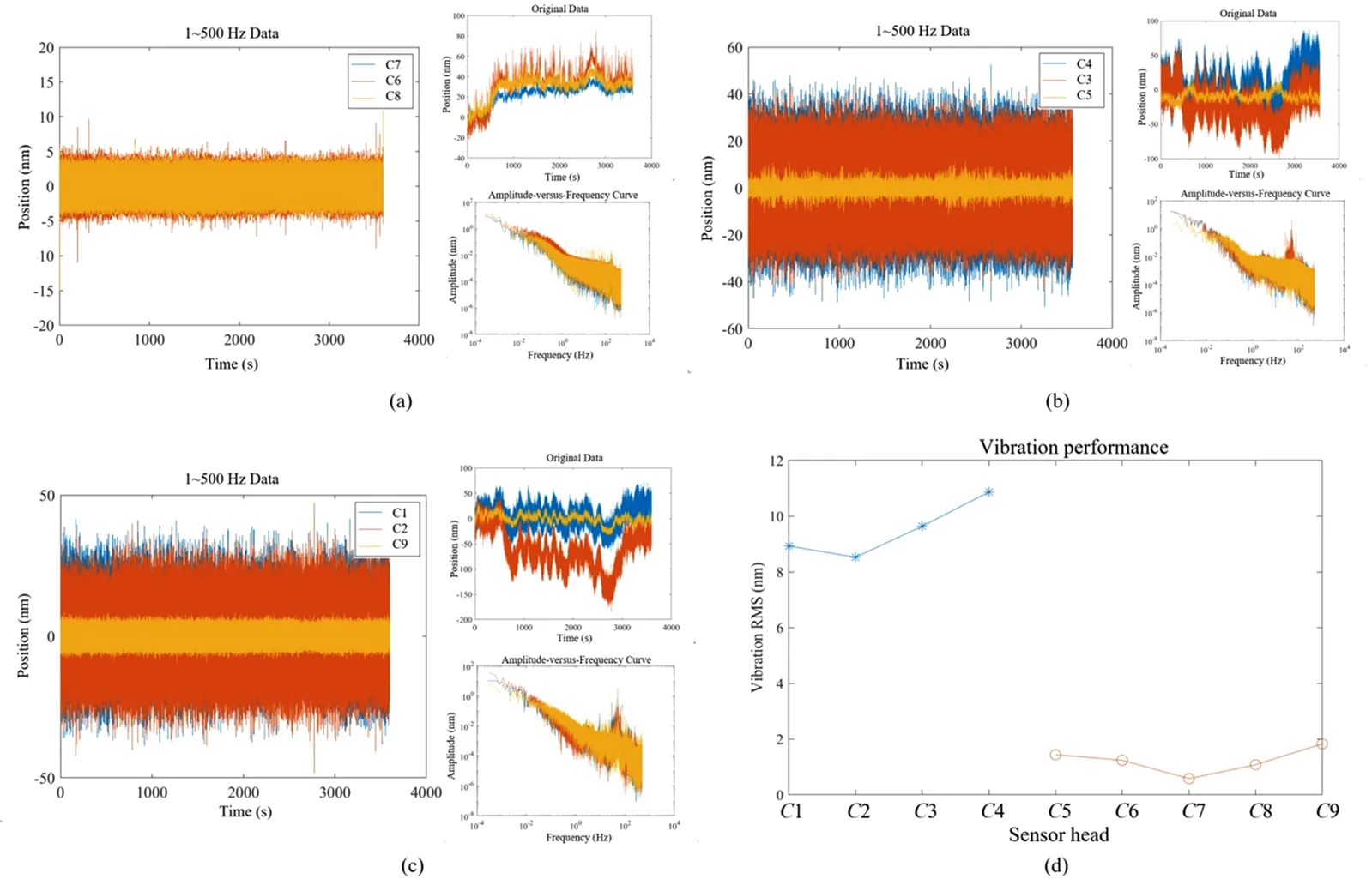
The 1 h stability measurement results of sensor heads *Ci* (*i* = 1,…, 9). The RMS summarized results are depicted in (*d*).

**Figure 14:**
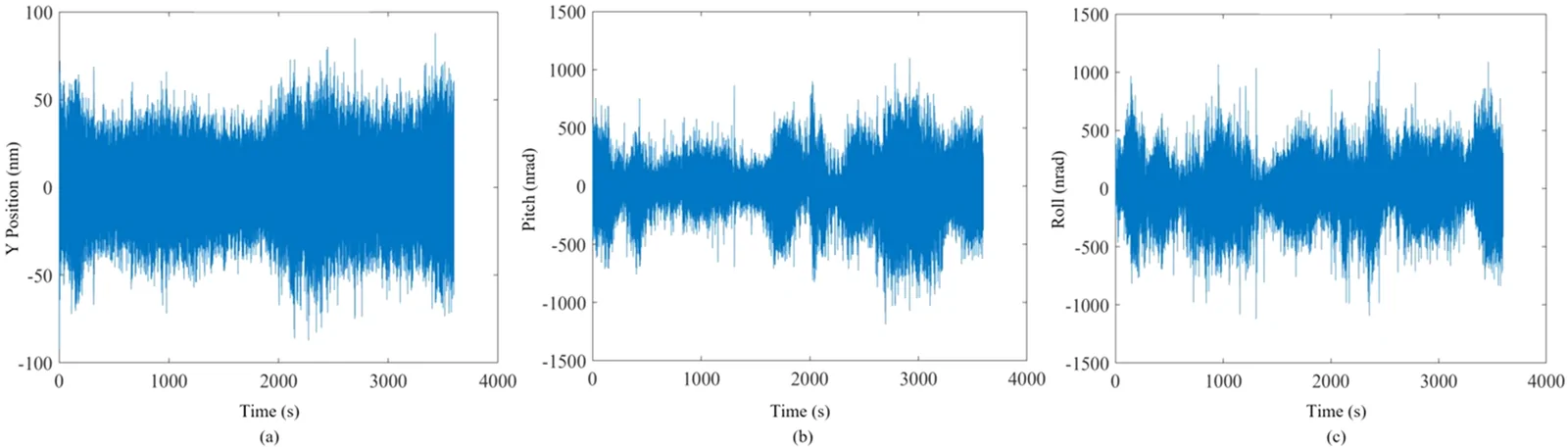
The 1 h stability of the chamber. Panel (*a*) is the *Y*-axis position stability, while panels (*b*) and (*c*) are the pitch and roll attitude stability.

Three Attocube 3010 interferometers host machines are used to obtain 1 h stability data, as shown in Fig. 13 ▸. The acquisition frequency is 1000 Hz, and the data of *Ci* (*i* = 1,…, 9) are illustrated in Figs. 13 ▸(*a*), 13(*b*) and 13(*c*). The left diagrams are the data after a high-pass filter (*i.e.* 1–500 Hz). The diagrams in the upper-right corner show the original 1 h data, and those in the lower-right show the amplitude-versus-frequency curves. The RMS of *C*7, *C*6, *C*8, *C*5 and *C*9, which monitor the sample, are 0.58 nm, 1.24 nm, 1.08 nm, 1.44 nm and 1.83 nm, respectively. The RMS of *C*4, *C*3, *C*1 and *C*2, which monitor the position of the AKB-mirrors, are 10.87 nm, 9.64 nm, 8.93 nm and 8.53 nm, respectively. These measurement values about the AKB-mirrors are obviously larger than those of the sample. According to Section 4.4, the measurement optical path passes through the sapphire window welded to the chamber, and the measurement shows notable sensitivity to chamber vibration. However, the direct experiments to evaluate the AKB-mirrors vibration in ID09 are not currently feasible as implementing a windowless configuration would necessitate disassembling the sample setup and venting the vacuum in the AKB vessel. As a result, the only way to clarify this issue is to measure the vibration of the chamber. Three sensor heads are arranged along the *Y*-axis (beam direction) so that the *Y*-axis, roll and pitch of the chamber can be measured and are shown in Fig. 14 ▸. Fig. 14 ▸(*a*) shows the average values of three sensor heads that express the *Y*-axis vibration. Pitch and roll vibration are shown in Figs. 14 ▸(*b*) and 14(*c*), respectively. The RMS values are 15.85 nm, 151.35 nrad and 156.06 nrad at 1–500 Hz. The measured coupling angle vibration of the chamber is about 220 nrad. According to equations (17) and (18), a 220 nrad coupling angle vibration causes a 7.86 nm OPD for HFM and 6.18 nm OPD for VFM. These results are consistent with the analysis in Section 4.4 and Table 2 ▸. Therefore, the chamber vibration accounts for the majority of the error and is larger than the vibration of the AKB-mirrors themselves. However, the whole mechanism is fed into the chamber through the bellows. The chamber and internal mechanism are decoupled in structure. And the AKB-mirrors configuration has a connective granite base with a sample setup. Therefore, it is safe to assume that the actual vibration of the AKB-mirrors configuration can be regarded as being of the same order of magnitude as that of the sample setup. To avoid introducing unnecessary errors, only the interferometer data for the sample (*C*1 to *C*4) were used in the ptychography experiments, excluding the interferometer data associated with the AKB-mirrors configuration (*C*5 to *C*9).

**Figure 15:**
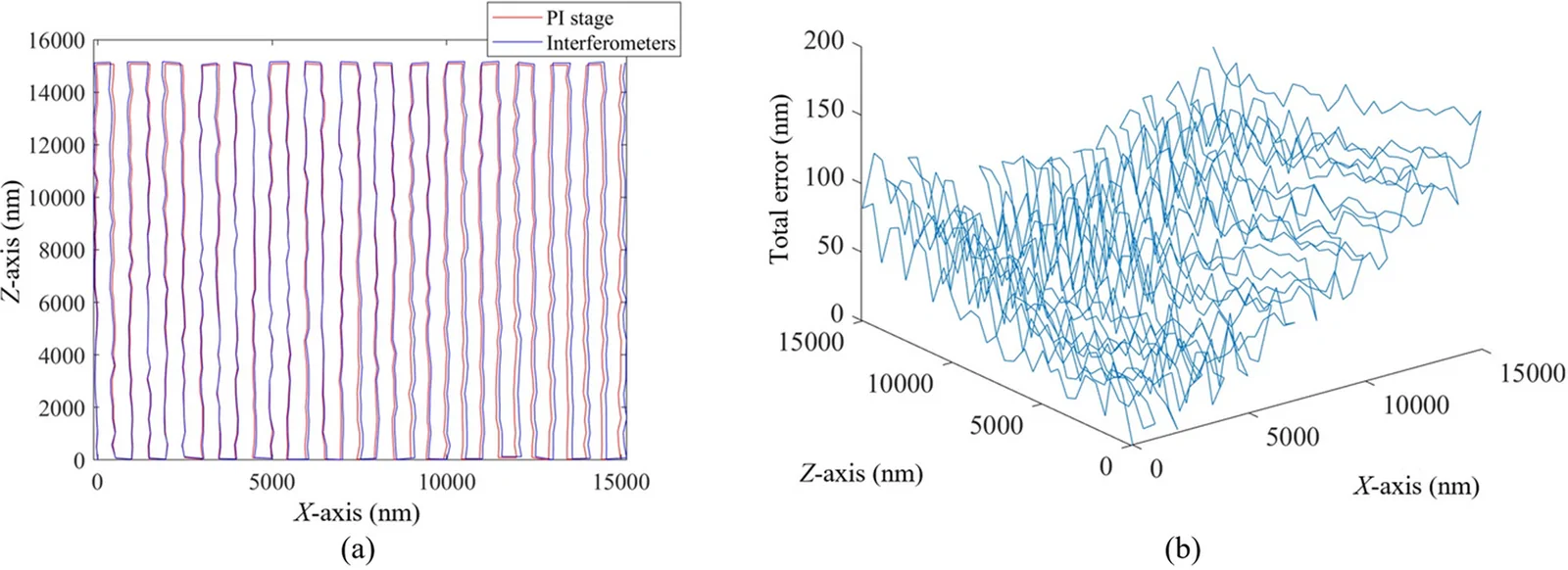
(*a*) Scanning trajectory from interferometers and piezo’s capacitive sensors integrated in the PI stage. (*b*) Difference between the interferometer values and the PI stage readout.

Finally, comparison experiments between interferometers and the piezo’s capacitive sensors integrated within the PI stage are provided to illustrate the performance of the interferometer setup. The data were reconstructed with a mixed-state algorithm, and the accuracy is refined by a position correlation algorithm (Dong *et al.*, 2026 ▸; Zhang *et al.*, 2013 ▸). but not the feedback control. The snake raster pattern generated by the interferometer values and PI stage readout is shown in Fig. 15 ▸(*a*), and the difference between interferometer values and the PI stage readout is shown in Fig. 15 ▸(*b*). The different slope trend between the long travel stroke (280 µm in calibration) and the short travel stroke (15 µm in snake raster pattern) is the main source of discrepancy between the piezo’s capacitive sensor and the interferometric measurement. In Fig. 15 ▸(*b*), the RMS position error between the piezo’s capacitive sensor and the interferometric measurement or scan step errors in Fig. 15 ▸(*b*) is 75.93 nm. The maximum position error value is 174.21 nm, and the minimum position error value is 1.91 nm.

**Figure 16:**
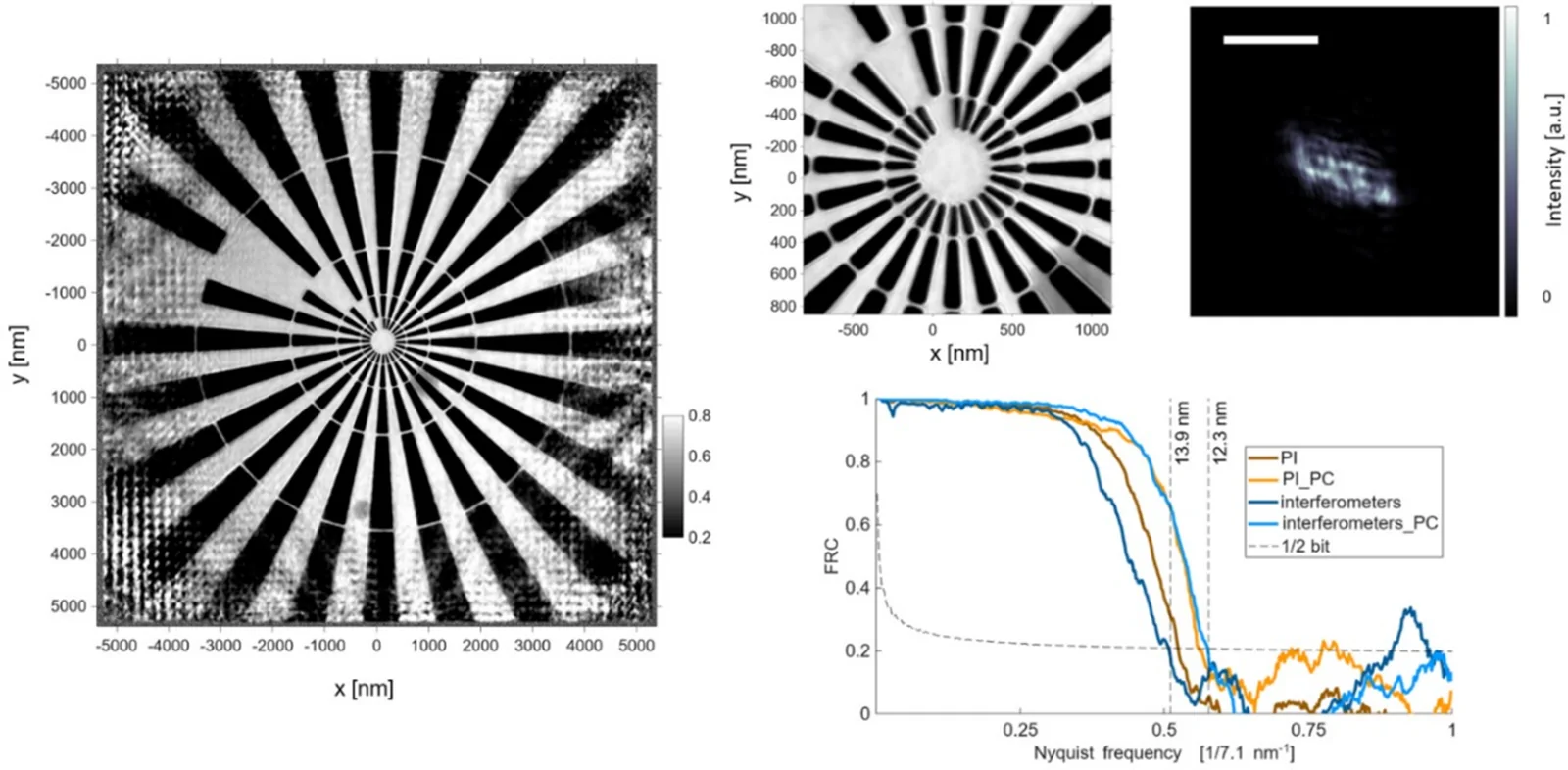
FRC resolution for the PI stage callback, the PI stage callback with position corrections, the interferometer system, and the interferometer system with position corrections are 13.9 nm, 12.7 nm, 14 nm and 12.3 nm, respectively. Scalebar: 2 µm.

The reconstruction resolution by ptychography is given. The resolution target is made of a silicon nitride coating with 600 nm gold. The innermost fringe has a width of 30 nm. The beam was slightly defocused (∼1 mm away). The beam size was ∼2 µm in diameter. The photon flux is ∼6 × 10^9^ photons s^−1^ @ 12.4 keV. The exposure is 1 s. The scan path follows a snake raster pattern (grid scan). The step size is ∼200 nm. The two object images were reconstructed from two separate datasets acquired with almost the same experimental conditions and parameters within 90 min. The reconstructions were carried out in the same computational environment with the same initial parameters. The detector type is Eiger 2 XE 4m from Dectris, characterized by 2162 pixels × 2068 pixels with 75 µm pixel size. The sample–camera distance is ∼9.8 m. The achievable spatial frequency is 1/7.1 nm^−1^. The spatial frequency cutoff used for phase retrieval is ∼1/7.5 nm^−1^, and the theoretically expected spatial resolution is ∼1/7.1 nm^−1^. A comparison between the interferometer values and the PI stage results is presented in Fig. 16 ▸. The FRC (Yin *et al.*, 2015 ▸) resolution for the PI stage callback, the PI stage callback with position corrections, the interferometer system, and the interferometer system with position corrections are 13.9 nm, 12.7 nm, 14 nm and 12.3 nm, respectively. The scalebar is 2 µm. After position refinements, both reconstructions based on interferometric displacement measurements and piezo’s capacitive sensors yielded high image quality, provided that the two positioning systems were carefully calibrated and aligned. Besides, the innermost 12 o’clock pattern of the Siemens star was significantly blurred. We are highly suspicious that it is caused by radiation damage, as this phenomenon was reproducible across both measurements.

## 6. Conclusion

A metrology method and error analysis for the HEPS ID09 ptychography setup are proposed and implemented. The proposed metrology method applies to a wider range of similar equipment. The detailed error analysis is specified for our setup, including asymmetric installation, Abbe error, mirror surface error, window vibration, and error about the cylinder retroflector. The metrology system, including nine sensor heads, is operating properly at the setup and contributes to the 2D ptychography experiment. The sample setup achieves a positional stability of less than 2 nm RMS from 1 Hz to 500 Hz. The key point of this paper is the interferometer metrology strategy. The implementation of 12.3 nm reconstruction resolution of ptychography verifies that the strategy and setup are available.

Though the resolution difference between the interferometer and the PI stage is marginal, the interferometric measurement remains necessary. First, the nonlinear coordinate scaling transformation in the piezo’s capacitive sensor integrated within the PI stage brings challenges to the position correction algorithm. The position correction algorithm can barely deal with the local tightly adjacent patterns. Second, the coupled motion relationships among the axes render the interferometer essential for trajectory correction in fly scan. The fly scan ptychography is impossible without interferometers. Third, the interferometer approach enables the measurement of the relative position between the focal spot and the sample. It is extremely difficult to perform alignment and measurement, either for a cylinder retroflector in a very confined space or for a cross-vessel configuration. Owing to the vibration of the chamber, the interferometer values for AKB-mirrors have not been included for ptychography up to now. It can be reasonably anticipated that even better performance could be achieved if the interferometer values for AKB-mirrors had been incorporated, for example, into the high-stiffness chamber design or the chamber vibration monitor. Although many aspects of our work still require further improvement, the current approach, where all sensors are mounted on a compact independent frame and measure the vibration of the focal spot perpendicular to the beam direction from the beam direction, has proven feasible.

Furthermore, further interferometer-based research should focus on fly scan and 3D ptychography. Interferometers can measure coupled motion relationships and are useful for trajectory correction. In addition, rotation errors in 3D experiments can only be corrected by external interferometers.

## Figures and Tables

**Table 1:** Parameter configuration

	HFM	VFM
Incidence angle	θ_*X*_ = 13°	θ_*Z*_ = 4°
Measurement arm	*L*_*X*_ = 150 mm	*L*_*Z*_ = 400 mm
Interferometer values	*C*1 = *C*2 = 100 nm	*C*3 = *C*4 = 100 nm
Atmosphere refractive indices	*n*_a_ = 1.0003	
Sapphire refractive indices	*n*_s_ = 1.74	
Vacuum refractive indices	*n*_v_ = 1	
Sapphire thickness	*D*_s_ = 2 mm	

**Table 2:** Error analysis values Installation asymmetry and some negligible terms are excluded from the total error budget.

Error term	HFM	VFM	Values of Δθ_*X*_ or Δθ_*Z*_	Definition of Δθ_*X*_ or Δθ_*Z*_
Asymmetric installation	1.80 nm	1.75 nm	1°	Installation angle errors
Abbe error	1.50 nm	4.00 nm	10 nrad	Angular stability
Mirrors surface error	<0.05 nm	<0.05 nm	1 µrad	Slope error
Window vibration error	3.57 nm	2.81 nm	100 nrad	Rotation variation
Total error	5.07 nm	6.81 nm		

**Table 3:** Absolute values of the error Δ*X*_S_ in relation to deflection radius *r* and rotation angle α Δ*X*_S_ values greater than 10 nm have been replaced by spaces

		α (°)						
		2	5	10	12	15	20	25
*r* (mm)	0.01	0.07	0.10	0.16	0.18	0.23	0.30	0.39
	0.02	0.09	0.16	0.31	0.38	0.51	0.75	1.02
	0.05	0.16	0.4	1.06	1.41	2.02	3.24	4.70
	0.08	0.24	0.77	2.27	3.09	4.53	7.49	
	0.10	0.31	1.07	3.32	4.57	6.77		
	0.12	0.39	1.43	4.59	6.33	9.45		
	0.15	0.51	2.05	6.85	9.52			

**Table 4:** Position accuracy and repeatability measurement results Position accuracy and repeatability of the *Z*-, *Y*- and *X*-axes are shown on the diagonal of the table. Other measurements express the results of straightness.

Position accuracy (nm) / straightness error (nm)	*Z*-axis	*Y*-axis	*X*-axis
*Z* stroke	**55.80**	40.36	66.12
*Y* stroke	4.31	**23.17**	18.37
*X* stroke	2.95	12.35	**24.94**

Repeatability (nm)			
*Z* stroke	**9.48**	2.23	2.95
*Y* stroke	2.08	**16.04**	14.20
*X* stroke	2.38	10.46	**18.82**
